# Influence of dual stratification on the magnetohydrodynamic flow of Jeffrey nanofluid over an exponentially stretching permeable sheet with viscous dissipation and Joule heating

**DOI:** 10.3389/fchem.2024.1451053

**Published:** 2025-02-10

**Authors:** M. Siva Sankari, M. Eswara Rao, Fuad A. Awwad, Emad A. A. Ismail, O. D. Makinde, Waris Khan

**Affiliations:** ^1^ Department of Mathematics, Saveetha School of Engineering, Saveetha Institute of Medical and Technical Sciences (SIMATS), Chennai, Tamil Nadu, India; ^2^ Department of Quantitative Analysis, College of Business Administration, King Saud University, Riyadh, Saudi Arabia; ^3^ Faculty of Military Science, Stellenbosch University, Saldanha, South Africa; ^4^ Department of Mathematics and Statistics, Hazara University, Mansehra, Pakistan

**Keywords:** Jeffery nanofluid, exponentially stretching sheet, viscous dissipation, Joule heating, double stratifications

## Abstract

Nanoparticles show superior potential for enhancing thermal properties compared to conventional particle–liquid suspensions. This investigation delves into magnetohydrodynamics (MHD) drift, heat, and mass transfer effects within a Jeffery nanoparticle liquid. The study includes transference equations that consider the influences of thermophoresis and Brownian motion on particle deposition. The analysis examines the impact of a nanofluid through a porous, exponentially elongating sheet, focusing on the double-stratification effects on heat and mass transference. The primary emphasis is on the formulated thermal energy equation, which incorporates Joule heating, heat generation, and ohmic dissipation terms. The initial step involves transforming the non-linear primary equations and their related boundary conditions into non-dimensional forms using similarity variables. The homotopy analysis method is then applied to obtain analytical results for the equations. Graphical representations of the impacts of various parameters on velocity and temperature values are presented, along with a detailed discussion of these impacts. A comprehensive analysis of specific parameters on the drag force factor-reduced Nusselt number and Sherwood number is provided and illustrated. Additionally, this research is applicable in environmental engineering, particularly in managing thermal pollution in water bodies, by aiding in predicting temperature distribution and the mixing behavior of effluents.

## Introduction

Nanofluids are examined for their potential applications in heat transfer, cooling systems, lubrication, and various industrial processes where enhanced thermal conductivity or other improved properties are desired. The interdisciplinary nature of the nanofluid field encompasses fluid dynamics, materials science, and thermal engineering. Khan and Pop, (2010) discussed the movement of nanofluids through a stretching sheet in the boundary layer. Sujata et al. (2023) explored the thermophysical properties of nanoliquids and their potential for enhancing heat transference. Ali et al. (2019a) explored the heat and mass transfer characteristics of 3D Maxwell nanofluid as it moves over an exponentially stretching surface.

Jeffery fluid is a viscoelastic liquid known for its anisotropic behavior, meaning it changes its rheological response and transmits shear stresses depending on the deformation rate. Widely used in studying complex fluids and rheology, the Jeffery fluid model is also applied in various fields, including geophysics, to understand behavior under diverse conditions. Samina et al. (2022) investigated phase portraits, multistability phenomena, and velocity profiles for nanofluids through the magnetohydrodynamics (MHD) Jeffery flow. The analytical method with the KKL nanoliquid model predicts multiple solutions for MHD Jeffery drift, as explored by Rana et al. (2019) on related heat transference problems.

The following sections discuss the impact of magnetic fields on liquid mechanics and dynamic energy in MHD. These sections also cover scenarios where such flows alter the ambient magnetic field. Utilizing MHD to control the motion of electrically conducting liquids offers potential benefits for pumps, propulsion systems, and other fluid-handling devices. Elboughdiri et al. (2023) investigated using the passive control method to simulate Jeffery nanofluid drifts with thermal augmentation near an impermeable suctioned surface, considering buoyancy and Lorentz force effects. Ali Abro et al. (2019) explored the thermal characteristics of MHD Jeffery liquid using contemporary non-integer-order derivatives in the analysis. Hussain et al. (2022) examined the sensitivity analysis of MHD nanofluid flow over an exponentially stretched surface with non-uniform heat flux through a response surface methodology.

A substance is recognized as a porous medium when it displays interconnected empty spaces, pores, or voids. Factors like pore size, shape, and connectivity influence this property. Porous media are commonly found in natural and engineered systems, such as soil, rocks, biological tissues, water filters, catalytic converters, and thermal insulation materials. Bilal et al. (2021) analyzed the dynamics of a chemical reaction in a porous medium made of Jeffery liquid, considering the influence of a magnetic field on a boundary layer under various slip conditions. Abd-Alla et al. (2023) observed the heat and mass transference characteristics of Jeffery fluid under peristaltic waves within a rotating frame involving porous media with chemically reactive species over a chemical process. Ali et al. (2024) performed a theoretical investigation of the unsteady MHD flow of Casson hybrid nanofluid in a porous medium, emphasizing the applications of thermal radiation and nanoparticles.

Exponential stretching is quantified by a parameter characterizing the rate of increase in the sheet’s length. Depending on various problems and boundary conditions, solutions to these mathematical models can provide helpful guidelines for understanding flow patterns and heat transfer characteristics across surfaces with exponential stretching. Thenmozhi et al. (2023) deliberated the inspiration of Jeffery fluid on MHD drift in heat transfer systems with elongated porous sheets. Reddappa and Sreenadh (2022) discovered the inspiration of double stratification on Jeffery fluid drift with electrical MHD, involving second-order chemical processes through an exponentially elongating sheet. Ali et al. (2021) explored the heat and mass transfer characteristics of 3D Oldroyd nanofluid as it moves over an exponentially stretching surface.

Joule heating is fundamental to many electrical devices and systems, such as electric heaters, toasters, incandescent lamps, and electronic components. Although it is often considered wasteful energy dissipation in some contexts, it is intentionally used for heating in others. Understanding Joule heating is crucial for managing the design and operation of electrical systems to prevent overheating and ensure efficiency. Al-Khaled et al. (2022) presented a mathematical model for analyzing the radiative peristaltic drift of Jeffery liquid in curved channels with ohmic heating. Harish Babu and Satya Narayana (2016) explored MHD Jeffery fluid flow through a power-law heat flux stretching sheet under Joule heating. Awais et al. (2022) conducted a numerical analysis of MHD axisymmetric rotating Bodewadt flow, considering the effects of viscous dissipation and ohmic heating.

Viscous dissipation is significant in fluid systems with substantial deformation or cutting, such as the pipe flow, liquid motion around obstacles, and fluid response to specific stresses. Understanding and measuring viscous dissipation is essential for designing and evaluating fluid systems, including their energy balance and heat distribution. Engineers may need to control this effect to improve efficiency and effectiveness. Li et al. (2022) studied Hall effects and ohmic dissipation under wave frame conditions for peristaltic Jeffery nanofluid transport. Al-Khaled et al. (2019) analyzed the influences of a heat source and chemical reactions on Jeffery fluid stagnation point flow, considering ohmic dissipation. Ashraf et al. (2022) investigated how viscous dissipation and magnetohydrodynamics influence periodic heat transfer along a cone in a porous medium.

Heat generation refers to creating thermal energy within a system or material, usually resulting from physical or chemical processes that convert other forms of energy into heat. Understanding and controlling heat generation are crucial in both natural and engineered systems. Dadhich et al. (2021) studied the thermally radiated drift of Jeffery fluids containing nanoparticles over a surface with varying thicknesses. Azhar et al. (2019) observed the influence of internal heat sources and chemical reactions on the stagnation point drift of Jeffery fluid, accounting for viscous dissipation. Ali et al. (2018) analyzed the three-dimensional MHD flow of Maxwell nanofluid containing gyrotactic microorganisms, factoring in the effects of heat sources and sinks.

Double stratification affects outcomes depending on the context in which it is applied. Stratification generally refers to the layering of different strata or layers. Understanding double stratification is vital for studying the dynamics of lake ecosystems, including nutrient cycling, oxygen distribution, and habitat preferences of various organisms. Muhammad et al. (2022) studied the bioconvective transfer of Jeffery nanofluid with gyrotactic motile microbes in a doubly stratified environment. Siva Sankari et al. (2023) observed the effects of dual stratification on Casson nanoliquid through an exponentially elongating sheet. Ali et al. (2019b) examined the effects of stratification in the inclined rheology of upper convected Maxwell (UCM) nanomaterials.

The novelty of the current analysis is highlighted through several aspects. First, it explores stratification effects in the MHD boundary layer-stretched flow of nanofluid-containing nanoparticles, incorporating Joule heating, viscous dissipation, heat generation, and thermal radiation—an area not previously investigated. Second, the study focuses on non-Newtonian fluids, specifically Jeffery nanofluid, further narrowing the research scope. Third, it examines the flow dynamics over an exponentially stretching sheet, adding complexity to the analysis. Fourth, convergent solutions of non-linear dimensionless expressions are developed using a homotopic procedure, ensuring robust and accurate results. The behaviors of various pertinent parameters on velocity, temperature, and concentration are thoroughly examined. The Nusselt and Sherwood numbers are computed and analyzed, offering valuable insights into the system’s thermal and mass transfer characteristics.

In various industrial and engineering applications, understanding the behavior of Jeffrey fluid over an exponentially stretching sheet is crucial, mainly when accounting for complex thermal and concentration effects. The study of such systems includes the impacts of viscous dissipation and Joule heating, which are essential in processes involving significant energy transformations. Additionally, heat generation within the fluid and the effects of thermal radiation are critical for accurate thermal management and optimization. Double stratification, incorporating both thermal and solutal stratification, further complicates the fluid behavior, making it necessary to consider these factors to achieve precise control and efficiency in applications such as polymer processing, cooling of electronic devices, and designing advanced material manufacturing processes.

### Mathematical formulation

We consider the two-dimensional MHD drift of Jeffrey nanofluid over an exponentially stretching sheet. Heat and mass transference impacts are taken into account. Standard to the drift direction is encountered an applied magnetic field of strength. $B_{0}$ . The concentration of the magnetized Jeffrey liquid is taken in the existence of the solutal stratification impact. The drift field is demonstrated by heat generation, thermal stratification, and thermal radiation impacts. The temperature drift regime is further strengthened by taking mixed convection features. In addition, viscous dissipation and Joule heating effects are also considered.

**FIGURE 1:**
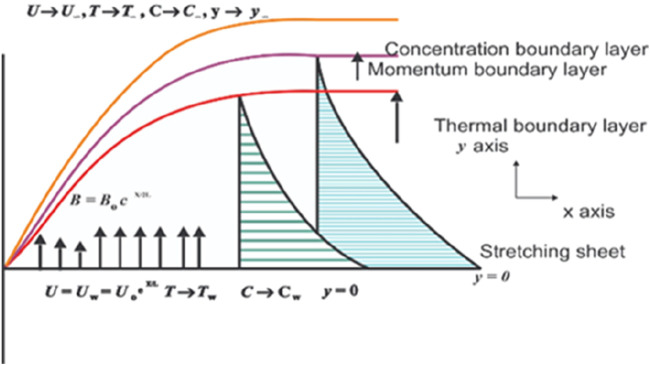
Geometry of the problem.

This investigation explores the boundary stratum drift of a liquid through an exponentially overextended pane in a porous medium within two extents. The y-axis is perpendicular to a varying magnetic field applied to the peripheral, where it is persistent, and the x-axis is assumed to be extended with velocity U. The implication is $T_{\infty }\left(x\right)=T_{0}+ce^{x/2L}$ . The variable quantities b, c, m, and n are considered optimistic if the position temperature $T_{0}$ and concentration are *C*_0_. The inconstant chemical variation rate of the second-order irrevocable procedure is $k_{r}=\frac{1}{2}\left(\frac{k_{0}}{m}\right)e^{x/2L}k_{0}$ , where $k_{0}$ is a persistent, and porousness is K′=k*exL , where k* is insistent. Figure 1 shows the drift regime’s thermal, species, and momentum boundary stratum.

divV=0, (1)

$$\rho \frac{dv}{dt}=\mathrm{div}\tau +\rho b.$$ (2)

The Cauchy stress tensor can be represented for a Jeffery fluid as (Ramzan et al., 2017)

τ=−pI+S, (3)

where the extra stress tensor S is defined as

$$S=\frac{\mu }{1+\lambda_{1}}\left[A_{1}+\lambda_{2}\left(\frac{\partial A_{1}}{\partial t}+\nabla .V\right)A_{1}\right].$$ (4)

The first Rivlin–Ericksen tensor is expressed as follows:

$$A_{1}=\left(\nabla V\right)+{\left(\nabla V\right)}^{t},$$ (5)

$$V=\left[u\left(x,y\right),v\left(x,y\right),0\right].$$ (6)

The Cauchy stress tensor is denoted by τ, the dynamic viscosity is represented by μ, $\lambda_{1}$ is the ratio of relaxation to retardation times, $\lambda_{2}$ is the retardation time, and $A_{1}$ is the Rivlin–Ericksen tensor. t represents matrix transpose, V velocity field.

The presented equations governing the drift are modeled as follows (Equations 9, 13, 25) (Reddappa and Sreenadh, 2022; Ramzan et al., 2017):

$$\frac{\partial u}{\partial x}+\frac{\partial v}{\partial y}=0,$$ (7)

$$u\frac{\partial u}{\partial x}+v\frac{\partial u}{\partial y}=\left(\frac{\upsilon }{1+\lambda_{1}}\right)\left(\frac{\partial^{2}u}{\partial y^{2}}+\lambda_{2}\left(\frac{\partial u}{\partial y}\frac{\partial^{2}u}{\partial x\partial y}+u\frac{\partial^{3}u}{\partial x\partial y^{2}}-\frac{\partial u}{\partial x}\frac{\partial^{2}u}{\partial y^{2}}+v\frac{\partial^{3}u}{\partial y^{3}}\right)\right)-\frac{\sigma B_{0}^{2}u}{\rho }-\left(\frac{\upsilon }{1+\lambda_{1}}\right)\frac{u}{k^{\prime }},$$ (8)

$$\left.\begin{array}{l} u\frac{\partial T}{\partial x}+v\frac{\partial T}{\partial y}=\alpha \frac{\partial^{2}T}{\partial y^{2}}+\frac{1}{\rho c_{p}}\frac{\partial q_{r}}{\partial y}+\frac{Q_{0}}{\rho c_{p}}\left(T-T_{\infty }\right)\\ +\tau \left[D_{B}\frac{\partial C}{\partial y}\frac{\partial T}{\partial y}+\frac{D_{T}}{T_{\infty }}{\left(\frac{\partial T}{\partial y}\right)}^{2}\right]+\frac{\sigma B_{0}^{2}u^{2}}{\rho c_{p}}+\left(\frac{\upsilon }{1+\lambda_{1}}\right)\frac{u^{2}}{k^{\prime }c_{p}}\\ +\frac{\upsilon }{c_{p}\left(1+\lambda_{1}\right)}{\left(\frac{\partial u}{\partial y}\right)}^{2}+\frac{\upsilon \lambda_{2}}{c_{p}\left(1+\lambda_{1}\right)}\frac{\partial u}{\partial y}\frac{\partial }{\partial y}\left(\frac{\partial u}{\partial x}+\frac{\partial v}{\partial y}\right) \end{array}\right\},$$ (9)

$$u\frac{\partial C}{\partial x}+v\frac{\partial C}{\partial y}=D_{B}\frac{\partial^{2}C}{\partial y^{2}}+\frac{D_{T}}{T_{\infty }}\frac{\partial^{2}T}{\partial y^{2}}.$$ (10)

The corresponding boundary restrictions are

u=U=U0exL, v=−Vx=−V0exL,T=Twx=T0+bex/2L,C=Cwx=C0+mex/2L, aty=0, (11)

$$u\to 0,T=T_{\infty }\left(x\right)=T_{0}+ce^{x/2L},C=C_{\infty }\left(x\right)=C_{0}+ne^{x/2L},\;asy\to \infty .$$ (12)

In this context, the reference velocity is represented by U_o_, the suction velocity is denoted by V(x) > 0, and the blowing velocity is denoted by V(x) < 0. The primary suction quality is indicated by > 0, while the initial blowing quality is indicated by < 0. Based on the Roseland approximation (Makinde and Animasaun, 2016) for radiative heat flux, $\partial q_{r}/\partial y\approx -\left(16\sigma *T_{\infty }^{3}/3k*\right)\partial^{2}T/\partial y^{2}$ , where k* is the mean absorption factor and ${nnotesotes\sigma }^{*}$ denotes the Stefan–Boltzmann persistent.

η=U02υLex2Ly, u=U0exLf′η,v=−υU02Lex2Lfη+ηf′η, θη=T−T∞Tω−T0,ϕη=C−C∞Cω−C0. (13)

The dimensionless forms of the equations for linear momentum, energy, and concentration, along with their corresponding boundary conditions, can be expressed as follows:

$$\left(\frac{1}{1+\lambda_{1}}\right)f^{\prime \prime \prime }+\left(1+\lambda_{1}\right)ff^{\prime \prime }-2\left(1+\lambda_{1}\right)f\prime^{2}+\frac{\beta }{\left(1+\lambda_{1}\right)}\left(\frac{3}{2}f{\prime \prime }^{2}-\frac{1}{2}ff^{\prime \prime \prime \prime }\right)-M\left(1+\lambda_{1}\right)f^{\prime }-Kp\left(1+\lambda_{1}\right)f^{\prime }=0,$$ (14)

$$\left.\begin{array}{l} \left(1+\frac{4}{3}K\right)\theta^{\prime \prime }+\mathrm{Pr}\left(f\theta^{\prime }-f^{\prime }\theta -Tf^{\prime }+Q\theta +Nb\theta^{\prime }\phi^{\prime }+Nt{\left(\theta^{\prime }\right)}^{2}\right)\\ +\mathrm{Pr}EcM{\left(f^{\prime }\right)}^{2}+\left(\frac{1}{1+\lambda_{1}}\right)\mathrm{Pr}Ec\left(f{\prime \prime }^{2}+\frac{\beta }{2}f^{\prime \prime }\left(3f^{\prime }f^{\prime \prime }-ff^{\prime \prime \prime }\right)\right)=0 \end{array}\right\},$$ (15)

$$\phi^{\prime \prime }+Sc\left(f\phi^{\prime }-f^{\prime }\phi -cf^{\prime }\right)+\left(\frac{Nt}{Nb}\right)\theta^{\prime \prime }=0.$$ (16)

The boundary conditions for the analyzed flow are

$$f^{\prime }=1,f=S,\theta =1-S_{t}\phi =1-S_{c},\text{as }\eta =0,$$ (17)

$$f^{\prime }\to 0,\theta \to 0,\phi \to 0\text{ as }\eta \to \infty .$$

Denoting the main as the variation concerning η, the various criticisms are represented as follows: the porous prarameter as $K_{P}=\frac{2L\upsilon }{k^{*}U_{0}}$ , the magnetic prarameter as $M=\frac{2\sigma B_{0}^{2}L}{\rho U_{0}}$ , the Prandtl number as $\mathrm{Pr}=\frac{\upsilon }{\alpha }$ , the heat source prarameter as Q, the thermally stratified structure as $S_{t}=\frac{c}{b}$ , the Schmidt number as $Sc=\frac{\upsilon }{D}$ , the chemically stratified prarameter as $S_{c}=\frac{n}{m}$ , the Eckert number as $Ec=\frac{U_{0}^{2}}{c_{p}\left(T_{w}-T_{\infty }\right)}$ , and the suction or blowing prarameter as $S=\frac{V_{0}}{\sqrt{\frac{\upsilon U_{0}}{2L}}}$ . For the suction prarameter, S > 0, while for the blowing prarameter, S < 0.

The critical physical extent of interest is the skin friction coefficient $C_{f}=\frac{\tau_{w}}{\rho U_{w}^{2}\left(x\right)}$ , local Nusselt number $\begin{array}{l} Nu=\frac{xq_{w}}{k\left(T_{w}-T_{\infty }\right)} \end{array}$ , and local Sherwood number $Sh=\frac{xJ_{w}}{D\left(C_{w}-C_{\infty }\right)}$ .

The symbols $\tau_{w}q_{w}J_{w}$ correspond to the shear stress, heat flux, and mass flux at the surface, respectively.

$$\tau_{w}=\left(\frac{\mu }{1+\lambda_{1}}\right){\left(\frac{\partial u}{\partial y}+\beta \frac{\partial u}{\partial y}\right)}_{y=0},\;{\;q}_{w}=-k{\left(\frac{\partial T}{\partial y}+\frac{16\sigma *T_{\infty }^{3}}{3k*k}\right)}_{y=0},J_{w}=-D{\left(\frac{\partial C}{\partial y}\right)}_{y=0}.$$ (18)

Utilizing Equation 7, the dimensionless drag force coefficient, as well as the rates of wall heat and mass transference, can be stated as follows:

2RexCfx=11+λ1f″0+βf″0x2L,NuxRex=−x2L1+43Kθ′01−T, ShxRex=−x2Lϕ′01−c. (19)

#### Solution procedure

To resolve Equations 5,–,7, below the boundary constraint Equation 8, we use the homotopy analysis method (HAM) with the resulting technique. The results take the assisting prarameter ℏ to change and resist the convergence of the explanations.

The primary presumptions are designated as

$$f_{0}\left(\eta \right)=1+S-e^{-\eta },\;\theta_{0}\left(\eta \right)=\left(1-S_{t}\right)e^{-\eta },\;\phi_{0}\left(\eta \right)=\left(1-S_{c}\right)e^{-\eta }.$$ (20)

The linear operatives are engaged as $L_{f},L_{\theta },L_{\phi },$

$$L_{f}\left(f\right)=\frac{d^{3}f}{d\eta^{3}}-\frac{df}{d\eta },\;L_{\theta }\left(\theta \right)=\frac{d^{2}\theta }{d\eta^{2}}-\theta ,\;L_{\phi }\left(\phi \right)=\frac{d^{2}\phi }{d\eta^{2}}-\phi ,$$ (21)

which have the following properties:

$$L_{f}\left(c_{1}+c_{2}e^{-\eta }+c_{3}e^{\eta }\right)=0,L_{\theta }\left(c_{4}e^{-\eta }+c_{5}e^{\eta }\right)=0,L_{\phi }\left(c_{6}e^{-\eta }+c_{7}e^{\eta }\right)=0.$$ (22)

In the overarching approach, $c_{i}\left(i=1-7\right)$ are the coefficients.

The consequent non-linear operators $N_{f},N_{\theta },N_{\phi },$ are provided as follows:

$$\begin{array}{l} N_{f}\left[f\left(\eta ;p\right)\right]=\left(\frac{1}{1+\lambda_{1}}\right)\frac{\partial^{3}f\left(\eta ;p\right)}{\partial \eta^{3}}+\left(1+\lambda_{1}\right)f\left(\eta ;p\right)\frac{\partial^{2}f\left(\eta ;p\right)}{\partial \eta^{2}}\\ -2\left(1+\lambda_{1}\right){\left(\frac{\partial f\left(\eta ;p\right)}{\partial \eta }\right)}^{2}+\frac{\beta }{\left(1+\lambda_{1}\right)}\left(\frac{3}{2}{\left(\frac{\partial^{2}f\left(\eta ;p\right)}{\partial \eta^{2}}\right)}^{2}-\frac{1}{2}f\left(\eta ;p\right)\frac{\partial^{4}f\left(\eta ;p\right)}{\partial \eta^{4}}\right)\\ -M\left(1+\lambda_{1}\right)\frac{\partial f\left(\eta ;p\right)}{\partial \eta }-Kp\left(1+\lambda_{1}\right)\frac{\partial f\left(\eta ;p\right)}{\partial \eta }, \end{array}$$ (23)

$$\begin{array}{l} N_{\theta }\left[f\left(\eta ;p\right),\theta \left(\eta ;p\right),\phi \left(\eta ;p\right)\right]=\left(1+\frac{4}{3}K\right)\frac{\partial^{2}\theta \left(\eta ;p\right)}{\partial \eta^{2}}\\ +\mathrm{Pr}\left(\begin{array}{l} f\left(\eta ;p\right)\frac{\partial \theta \left(\eta ;p\right)}{\partial \eta }-\frac{\partial f\left(\eta ;p\right)}{\partial \eta }\theta \left(\eta ;p\right)-S_{t}\frac{\partial f\left(\eta ;p\right)}{\partial \eta }+\\ Q\theta \left(\eta ;p\right)+Nb\frac{\partial \theta \left(\eta ;p\right)}{\partial \eta }\frac{\partial \phi \left(\eta ;p\right)}{\partial \eta }+Nt{\left(\frac{\partial \theta \left(\eta ;p\right)}{\partial \eta }\right)}^{2} \end{array}\right)+\mathrm{Pr}EcM{\left(\frac{\partial f\left(\eta ;p\right)}{\partial \eta }\right)}^{2}+\mathrm{Pr}Ec\left({\frac{1}{1+\lambda }}_{1}\right)\\ \left[{\left(\frac{\partial^{2}f\left(\eta ;p\right)}{\partial \eta^{2}}\right)}^{2}+\frac{\beta }{2}\frac{\partial^{2}f\left(\eta ;p\right)}{\partial \eta^{2}}\left(\begin{array}{l} 3\frac{\partial f\left(\eta ;p\right)}{\partial \eta }\frac{\partial^{2}f\left(\eta ;p\right)}{\partial \eta^{2}}-\\ f\left(\eta ;p\right)\frac{\partial^{3}f\left(\eta ;p\right)}{\partial \eta^{3}} \end{array}\right)\right], \end{array}$$

$$\begin{array}{l} N_{\phi }\left[f\left(\eta ;p\right),\theta \left(\eta ;p\right),\phi \left(\eta ;p\right)\right]=\frac{\partial^{2}\phi \left(\eta ;p\right)}{\partial \eta^{2}}+\left(\frac{Nt}{Nb}\right)\frac{\partial^{2}\theta \left(\eta ;p\right)}{\partial \eta^{2}}\\ +Sc\left(f\left(\eta ;p\right)\frac{\partial \phi \left(\eta ;p\right)}{\partial \eta }-\frac{\partial f\left(\eta ;p\right)}{\partial \eta }\phi \left(\eta ;p\right)-S_{c}\frac{\partial f\left(\eta ;p\right)}{\partial \eta }\right), \end{array}$$ (24)

The essential perception of the HAM is elucidated by Khan and Pop (2010); Sujata et al. (2023); Ali et al. (2019a); and Samina et al. (2022), and the following constitute the zeroth-order problems in Equations 5–7:

$$\left(1-p\right)L_{f}\left[f\left(\eta ;p\right)-f_{0}\left(\eta \right)\right]=p\hbar_{f}N_{f}\left[f\left(\eta ;p\right)\right],$$ (25)

$$\left(1-p\right)L_{\theta }\left[\theta \left(\eta ;p\right)-\theta_{0}\left(\eta \right)\right]=p\hbar_{\theta }N_{\theta }\left[f\left(\eta ;p\right),\theta \left(\eta ;p\right),\phi \left(\eta ;p\right)\right],$$ (26)

$$\left(1-p\right)L_{\phi }\left[\phi \left(\eta ;p\right)-\phi_{0}\left(\eta \right)\right]=p\hbar_{\phi }N_{\phi }\left[f\left(\eta ;p\right),\theta \left(\eta ;p\right),\phi \left(\eta ;p\right)\right].$$ (27)

The comparable restrictions for boundaries are as follows:

$$\begin{array}{l} {\left.f\left(\eta ;p\right)\right|}_{\eta =0}=S,{\left.\frac{df\left(\eta ;p\right)}{d\eta }\right|}_{\eta =0}=1,{\left.\theta \left(\eta ;p\right)\right|}_{\eta =0}=1-S_{t},{\left.\phi \left(\eta ;p\right)\right|}_{\eta =0}=1-S_{c},\\ {\left.\frac{df\left(\eta ;p\right)}{d\eta }\right|}_{\eta \to \infty }=0,{\left.\theta \left(\eta ;p\right)\right|}_{\eta \to \infty }=0,{\left.\phi \left(\eta ;p\right)\right|}_{\eta \to \infty }=0, \end{array}$$ (28)

where p $\in \left[0,1\right]$ is the embedding parameter and $\hbar_{f},\hbar_{\theta },\hbar_{\phi },$ are used to control the convergence of the solution. When p = 0 and p = 1, we have

$$f\left(\eta ;1\right)=f\left(\eta \right),\theta \left(\eta ;1\right)=\theta \left(\eta \right),\phi \left(\eta ;1\right)=\phi \left(\eta \right).$$ (29)

Upward $f\left(\eta ;p\right),\theta \left(\eta ;p\right),\phi \left(\eta ;p\right),$ in Taylor’s series about p=0

$$\begin{array}{l} f\left(\eta ;p\right)=f_{0}\left(\eta \right)+\sum_{m=1}^{\infty }f_{m}\left(\eta \right)p^{m},\theta \left(\eta ;p\right)=\theta_{0}\left(\eta \right)+\sum_{m=1}^{\infty }\theta_{m}\left(\eta \right)p^{m},\\ \phi \left(\eta ;p\right)=\phi_{0}\left(\eta \right)+\sum_{m=1}^{\infty }\phi_{m}\left(\eta \right)p^{m}, \end{array}$$ (30)

where

$$f_{m}\left(\eta \right)={\left.\frac{1}{m!}\frac{\partial f\left(\eta ;p\right)}{\partial \eta }\right|}_{p=0},\theta_{m}\left(\eta \right)={\left.\frac{1}{m!}\frac{\partial \theta \left(\eta ;p\right)}{\partial \eta }\right|}_{p=0},\phi_{m}\left(\eta \right)={\left.\frac{1}{m!}\frac{\partial \phi \left(\eta ;p\right)}{\partial \eta }\right|}_{p=0}.$$ (31)

By selecting the secondary constraints so that the series Equation 21 switches in Equation 20 and converges, we obtain

$$f\left(\eta \right)=f_{0}\left(\eta \right)+\sum_{m=1}^{\infty }f_{m}\left(\eta \right),\theta \left(\eta \right)=\theta_{0}\left(\eta \right)+\sum_{m=1}^{\infty }\theta_{m}\left(\eta \right),\phi \left(\eta \right)=\phi_{0}\left(\eta \right)+\sum_{m=1}^{\infty }\phi_{m}\left(\eta \right).$$ (32)

The following is satisfied by the mth-order problem:

$$\begin{array}{l} L_{f}\left[f_{m}\left(\eta \right)-\omega_{m}f_{m-1}\left(\eta \right)\right]=\hbar_{f}R_{m}^{f}\left(\eta \right),L_{\theta }\left[\theta_{m}\left(\eta \right)-\omega_{m}\theta_{m-1}\left(\eta \right)\right]=\hbar_{\theta }R_{m}^{\theta }\left(\eta \right),\\ L_{\phi }\left[\phi_{m}\left(\eta \right)-\omega_{m}\phi_{m-1}\left(\eta \right)\right]=\hbar_{\phi }R_{m}^{\phi }\left(\eta \right), \end{array}$$ (33)

The related conditions for boundaries are as follows:

$$\begin{array}{l} f_{m}\left(0\right)=f_{m}^{\prime }\left(0\right)=\theta_{m}\left(0\right)=\phi_{m}\left(0\right)=0\\ f_{m}^{\prime }\left(\infty \right)=\theta_{m}\left(\infty \right)=\phi_{m}\left(\infty \right)=0 \end{array}.$$ (34)

Here,

$$\begin{array}{l} R_{m}^{f}\left(\eta \right)=\left(\frac{1}{1+\lambda_{1}}\right)f_{m-1}^{\prime \prime \prime }+\left(1+\lambda_{1}\right)\sum_{k=0}^{m-1}f_{m-1-k}f_{k}^{\prime \prime }-2\left(1+\lambda_{1}\right)\sum_{k=0}^{m-1}f_{m-1-k}^{\prime }f_{k}^{\prime }\\ -M\left(1+\lambda_{1}\right)f_{m-1}^{\prime }-K_{p}\left(1+\lambda_{1}\right)f_{m-1}^{\prime }+\frac{\beta }{\left(1+\lambda_{1}\right)}\left(\frac{3}{2}\sum_{k=0}^{m-1}f_{m-1-k}^{\prime \prime }f_{k}^{\prime \prime }-\frac{1}{2}\sum_{k=0}^{m-1}f_{m-1-k}f_{k}^{⁗}\right), \end{array}$$ (35)

$$\begin{array}{l} R_{m}^{\theta }\left(\eta \right)=\left(1+\frac{4}{3}K\right)\theta_{m-1}^{\prime \prime }+\mathrm{Pr}EcM\sum_{k=0}^{m-1}f_{m-1-k}^{\prime }f_{k}^{\prime }+\\ \mathrm{Pr}\left(\sum_{k=0}^{m-1}f_{m-1-k}\theta_{k}^{\prime }-\sum_{k=0}^{m-1}f_{m-1-k}^{\prime }\theta_{k}-S_{t}f_{m-1}^{\prime }+Q\theta_{m-1}+Nb\sum_{k=0}^{m-1}\theta_{m-1-k}^{\prime }\phi_{k}^{\prime }+Nt\sum_{k=0}^{m-1}\theta_{m-1-k}^{\prime }\theta_{k}^{\prime }\right)\\ +\left(\frac{1}{1+\lambda_{1}}\right)\mathrm{Pr}Ec\left(\sum_{k=0}^{m-1}f_{m-1-k}^{\prime \prime }f_{k}^{\prime \prime }+\frac{\beta }{2}\left(3\sum_{k=0}^{m-1}f_{m-1-k}^{\prime }\sum_{l=0}^{k}f_{k-l}^{\prime \prime }f_{l}^{\prime \prime }-\sum_{k=0}^{m-1}f_{m-1-k}\sum_{l=0}^{k}f_{k-l}^{\prime \prime }f_{l}^{\prime \prime \prime }\right)\right), \end{array}$$ (36)

$$R_{m}^{\phi }\left(\eta \right)=\phi_{m-1}^{\prime \prime }+\left(\frac{Nt}{Nb}\right)\theta_{m-1}^{\prime \prime }+Sc\left(\sum_{k=0}^{m-1}f_{m-1-k}\phi_{k}^{\prime }-\sum_{k=0}^{m-1}f_{m-1-k}^{\prime }\phi_{k}-S_{c}f_{m-1}^{\prime }\right),$$ (37)

where

$$\omega_{m}=\left\{\begin{array}{l} 0,\text{if }p\leq 1\\ 1,\text{if }p>1 \end{array}\right..$$

The following are the general solutions:

$$\begin{array}{c} L_{f}\left(\eta \right)=f_{m}^{*}\left(\eta \right)+c_{1}+c_{2}e^{-\eta }+c_{3}e^{\eta }\\ \theta_{m}\left(\eta \right)=\theta_{m}^{*}\left(\eta \right)+c_{4}e^{-\eta }+c_{5}e^{\eta }\\ \phi_{m}\left(\eta \right)=\phi_{m}^{*}\left(\eta \right)+c_{6}e^{-\eta }+c_{7}e^{\eta } \end{array},$$ (38)

where $f_{m}^{*}\left(\eta \right),\theta_{m}^{*}\left(\eta \right),\phi_{m}^{*}\left(\eta \right)$ are the particular solutions. Mathematica is used to address linear homogeneous Equations 33, 34 in a consecutive manner of m = 1, 2, 3.

### Convergence of the HAM solution

**FIGURE 2:**
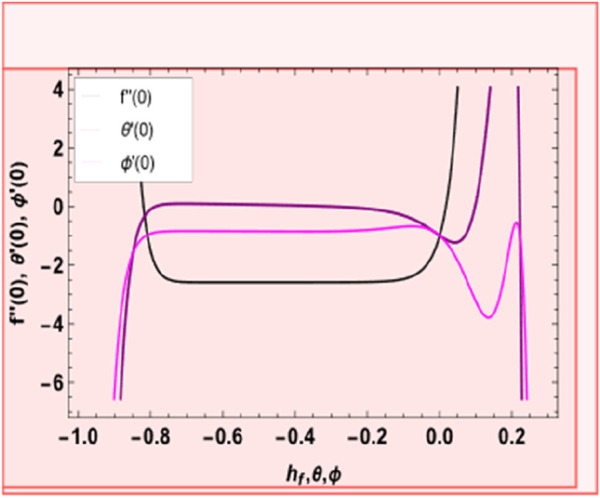
H-curve for function $f^{\prime \prime }\left(0\right),\theta^{\prime }\left(0\right),\phi^{\prime }\left(0\right)$ .

**FIGURE 3:**
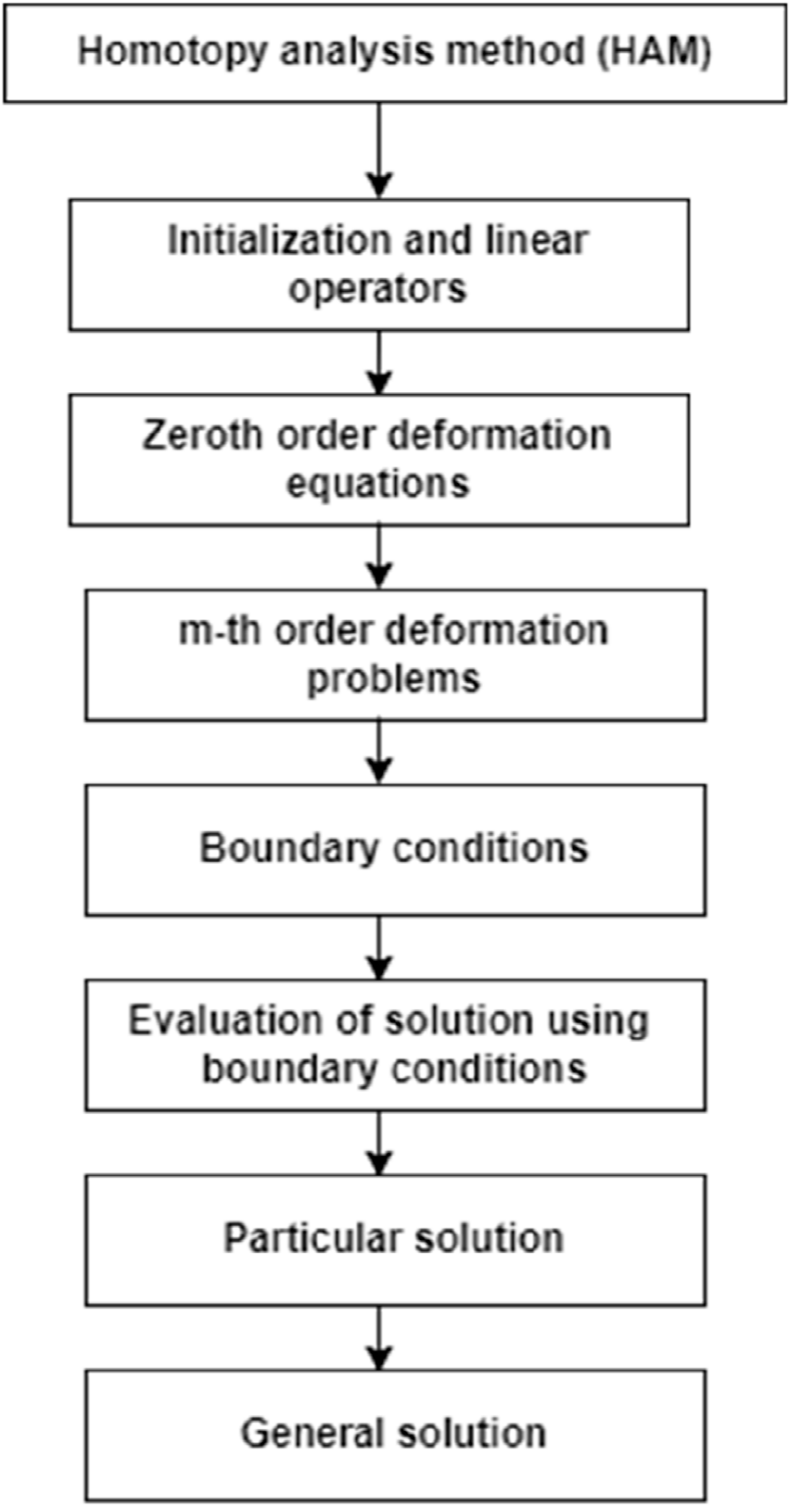
Flowchart of the HAM.

The equation series expansions provide the solutions. H-curves at the 18th order of approximation are shown in Figure 2, which helps pick up suitable values of $h_{\phi }$ . Figure 3 shows that the correct values of ℏ*f* are $-0.8\leq h_{f}\leq -0.1,-0.82\leq h_{\theta }\leq -0.12$ and $-0.73\leq h_{\phi }\leq -0.1$ .

### Convergent table

Table 1 is given to ensure solution convergence. This table clearly shows that convergence is obtained at the 48th order of approximations.

**TABLE 1:** HAM solution convergence at various approximation orders.

M	$f^{\prime \prime }\left(0\right)$	$\theta^{\prime }\left(0\right)$	$\phi^{\prime }\left(0\right)$
4	−1.22139	−0.78096	−0.707819
8	−1.3250	−0.693755	−0.584240
12	−1.37449	−0.628956	−0.50381 2
16	−1.39876	−0.579765	−0.450614
20	−1.4113	−0.549421	−0.419513
24	−1.42952	−0.526218	−0.396782
28	−1.43835	−0.507231	−0.370321
32	−1.44952	−0.496429	−0.358951
36	−1.45126	−0.489532	−0.349825
40	−1.45334	−0.487539	−0.337423
44	−1.45426	−0.486943	−0.336732
48	−1.45432	−0.486694	−0.336087
50	−1.45432	−0.486694	−0.336087

### Validation

**FIGURE 4:**
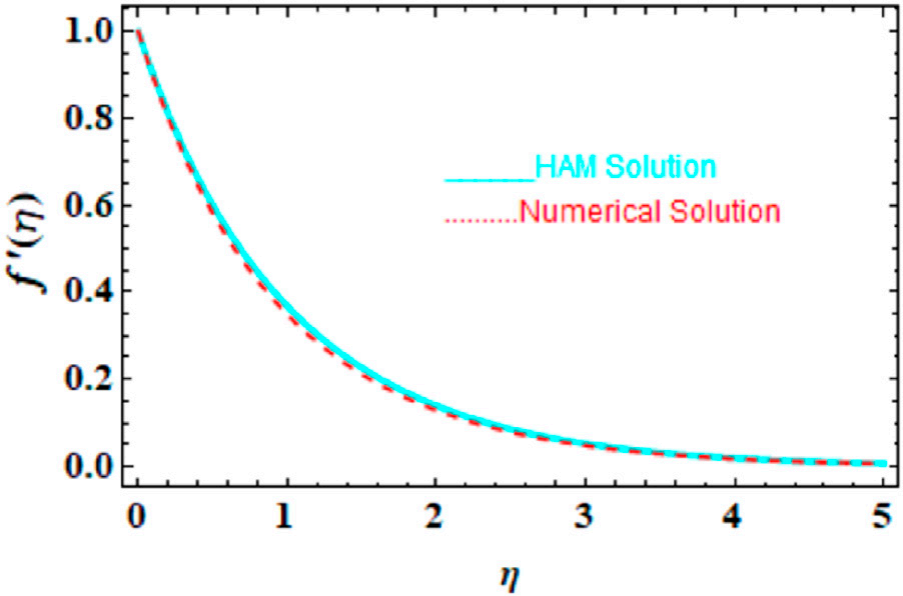
HAM and numerical comparison of the velocity profile $f^{\prime }\left(\eta \right)$ .

**FIGURE 5:**
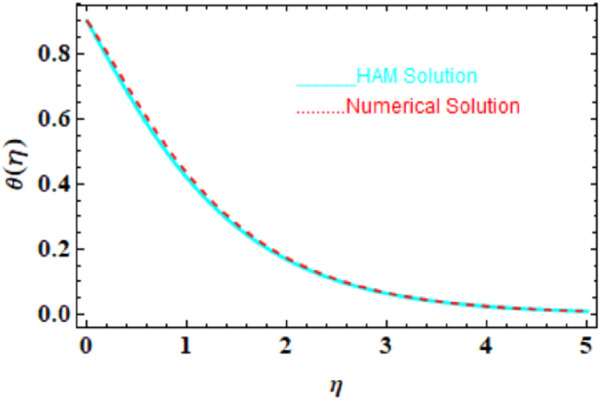
HAM and numerical comparison for the temperature profile $\theta \left(\eta \right)$ .

**FIGURE 6:**
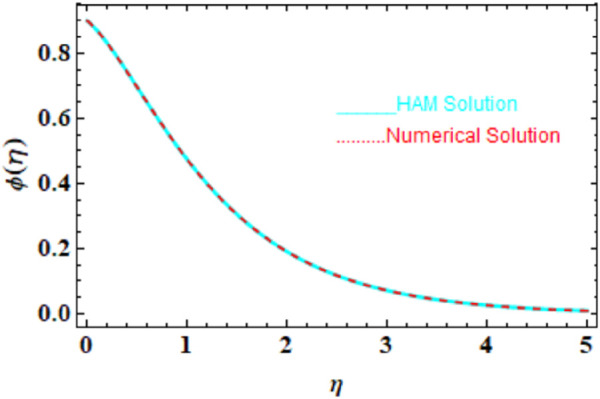
HAM and numerical comparison of the concentration profile $\phi \left(\eta \right)$ .

We compared the numerical solutions for velocity, temperature, and concentration graphs to validate our calculations with the HAM solutions, as shown in Figures 4–6. The comparisons demonstrate good agreement. Additionally, we compared our numerical results with the HAM solutions in Tables 2, 3; Supplementary Table S1. These comparisons confirm the accuracy of our calculation method.

**TABLE 2:** HAM and numerical comparison for the velocity profile $f^{\prime }\left(\eta \right)$ .

η	HAM solution	Numerical solution	Absolute error
0.0	1.000000	1.000000	0.000000
0.5	0.599264	0.598658	0.001213
1.0	0.367317	0.366,674	0.001286
1.5	0.226250	0.225743	0.001013
2.0	0.139053	0.138699	0.000709
2.5	0.085149	0.084916	0.000466
3.0	0.051976	0.051827	0.000297
3.5	0.031654	0.031561	0.000185
4.0	0.019248	0.019191	0.000114
4.5	0.011693	0.011658	0.000070
5.0	0.007099	0.007078	0.000043

**TABLE 3:** HAM and numerical comparison for the velocity profile $f^{\prime }\left(\eta \right)$ .

η	HAM solution	Numerical solution	Absolute error
0.0	0.900,000	0.900,000	0.000000
0.5	0.631487	0.632116	0.001257
1.0	0.417822	0.418306	0.000968
1.5	0.267020	0.267320	0.000600
2.0	0.167141	0.167318	0.000353
2.5	0.103325	0.103428	0.000206
3.0	0.063396	0.063456	0.000122
3.5	0.038720	0.038757	0.000072
4.0	0.023585	0.023606	0.000043
4.5	0.014342	0.014354	0.000026
5.0	0.008712	0.008720	0.000016

## Result and discussion

Different constraints were used to obtain the numerical values of local variables given in Supplementary Tables S2–S4. Supplementary Table S2 also shows that, at higher M, β, λ1, and Kp, there is an increase in the drag force factor. The results for −θ′(0), referred to as the local Nusselt number, are given in Supplementary Table S3. Conversely, as observed, while high Pr, Q, and Ec increase KTs, leading to improved heat transfer rate, this is not so for Supplementary Table S3. Sc, Nb, and Kp can be calculated against the Sherwood number *versus* Nt and Nb in Supplementary Table S4. Therefore, higher Sc, Nb, and Kp values enhance the mass transfer rate, while an increase in the mass transfer rate with an increasing Nt value is unlikely to occur.

### Velocity distribution

Supplementary Figure S1 in this paper helps us understand how magnetic field fluctuations affect velocity distributions. When the strength of the magnetic field increases, a drag force slows down the fluid’s motion. Stabilization can be achieved by applying a controlled transverse magnetic field, which helps delay from laminar to turbulent flow in the boundary layer flow. With Jeffery fluid having both elastic and viscous properties, this determines the direction and magnitude of the applied magnetic field. Such an interaction changes how fluid particles move, which leads to possible complicated MHD consequences that are guided by fluid conductivity and susceptibility to magnetism. The magnetohydrodynamic effect may become severe at higher values of these parameters concerning alignment with or against the fluid flow direction. The accurate control and understanding of such parameters are highly essential for optimizing process efficiency, as well as operation cost in applications like electromagnetic stirring in metallurgy, magnetic drug targeting in biomedicine, and aerospace engineering, where precise manipulation of the distribution of velocities is necessary for maximum operational effectiveness and performance improvement.

Supplementary Figure S2 shows an increase in the retardation time, λ2, which is linked to an increase in the Deborah number β. β relies on λ2; thus, whenever we increase this value, it means that the retardations will last longer. In physics, increasing the retardation time makes objects more elastic. Elasticity and viscosity effects are inversely proportional; hence, a decrease in viscosity leads to a higher fluid velocity. The velocity also increases when the Deborah number increases, as observed here. The complex and non-uniform flow patterns of Jeffery fluid are caused by its high Deborah number, which indicates more elastic behavior. Conversely, a lower Deborah number implies smoother and more uniform velocity distributions due to a more viscous behavior being exhibited. It is important to note that understanding how these fluids behave in polymer processing, biological flows, and other complex applications is necessary to optimize their final results or efficiency.

The porosity parameter Kp is investigated in Supplementary Figure S3 for velocity distribution. In Jeffery fluid, the porosity parameter significantly affects the flow by determining the proportion of void spaces present within the fluid structure. For instance, an increase in fluid porosity results in a decrease in its velocity, which is consistent with reality, as we know it. Moreover, this variation occurs due to official scenarios where increasing voids imply the reduced speed of a given material or liquid substance when it flows through it. Finally, beyond certain distances from the surface, boundary porosity has no impact on fluid motion at all. To control the product efficiency and enhance performance levels, filtration processes require knowledge about what happens to pore medium fluids and composite manufacturing.

Supplementary Figure S4 shows the impact of velocity, denoted by the ratio between relaxation and retardation time ${\;\lambda }_{1}$ . An increase in ${\;\lambda }_{1}$ indicates a longer relaxation time and a shorter retardation time. Consequently, fluid particles take more time to return to equilibrium after they are disturbed. The ratio of relaxation to retardation time in a Jeffery fluid is essential for determining its velocity distribution because it exhibits a viscoelasticity nature, as discussed earlier. On one hand, the relaxation time reveals how fast the fluid returns to its original shape when stress is removed, indicating elasticity; on the other hand, the retardation time indicates how rapidly the fluid deforms under stress meaning viscosity. On this note, higher ratios depict more elastic behavior with slow response times, while lower ratios indicate more viscous characteristics with fast velocity adjustments.

### Temperature distribution

The thermal radiation concept in the temperature field is shown in Supplementary Figure S5. The fluid’s internal energy increases with the increase in radiative parameters. In this way, increasing this parameter reduces the internal energy and the rate at which heat is transferred on the surface. These decreases in the rates of heat transfer can result in an increase in the temperature field since it occurs when the radiation’s variable increases. Consequently, varying the radiation parameter leads to a more significant temperature field. Understanding these outcomes of thermal radiation is very important for situations where Jeffery fluid is used mainly in high-temperature processes or space applications, and correct forecasting and control over temperature distributions are vital.

Supplementary Figure S6 shows how the temperature flows are distributed after heating. It was observed that with an increasing heat generation parameter, a corresponding increment also occurs in the temperature distribution within the boundary layer. The location of heat within Jeffery fluid mainly changes its temperature distribution by increasing the thermal energy content. Furthermore, outside sources of energy also cause additional heat input. Such activities as industrial processes and thermal management systems should know how heating affects heat gradients. This enables them to optimize their efficiency, producing quality outcomes because it can control them accurately.

Supplementary Figure S7 shows the impact of the Prandtl number on temperature distribution. This ratio is called the Prandtl number, which is defined as the ratio between momentum and thermal diffusivity. A high Prandtl number indicates that momentum diffusivity surpasses thermal diffusivity, thereby leading to modifications in fluid transport properties, such as increased heat capacity. Thus, an increase in the Prandtl number results in a decrease in temperature distribution. Understanding how fluctuations in Prandtl numbers affect industrial process cooling systems or environmental evaluations is vital because the accurate control of temperature gradients for optimization is essential for improved productivity and reliability.

The temperature variation concerning the Eckert number (Ec) is shown in Supplementary Figure S8. The thermal boundary-layer thickness and temperature increase as the Eckert number increases. The friction forces causing an increase in the Eckert numbers store heat energy within the fluid, which enhances the temperature profile. Conversely, smaller Eckert numbers indicate more even temperature profiles since conduction prevails over other mechanisms in these cases. In industrial processes that seek maximum efficiency and quality production, exact temperature regulation is desired; hence, understanding how the Eckert number works is essential here.

Supplementary Figure S9 shows the influence of the thermal stratification parameter on the temperature field. The difference in temperature between the surface and surrounding environment is referred to as thermal stratification. With the increase in thermal stratification parameters, the temperature field decreases, which is due to the temperature difference acting as the driving force. An increased temperature difference leads to a higher heat transfer rate. Therefore, a change in thermal stratification parameters would cause a reduction in temperature fields. Understanding the implication of this parameter is essential in areas including environmental monitoring, industrial processes, and material processing, where the exact control of such gradients is essential for optimal efficiency and quality assurance.

The dimensionless thermal profile is shown in Supplementary Figure S10 in response to variations in the Brownian motion parameter. At increased Nb, we observe an increase in θ(η) and the thermal boundary-layer thickness of the fluid flow. The phenomenon produces colliding particles that cause a heat transfer between them as they interact with this viscous–elastic Jeffrey fluid due to the Brownian motion. Those collisions lead to localized temperature changes and heat diffusion throughout the liquid, thus contributing to more homogeneous heat distribution. For example, in cases where Jeffery fluids are used as coolants in industrial processes or material manufacturing, elaborate management should be put up for thermal/heat issues requiring comprehension of how Brownian movement can individually influence temperature distribution patterns.

Supplementary Figure S11 shows the impact of the thermal motion parameter (Nt) on the temperature profile. The increase in Nt enhances θ(η), thereby thickening the thermal boundary layer. Its importance is such that Jeffery fluid exhibits critical influence, taking hot particles to cold places and redistributing heat energy all over its mass. This migration causes cooling in specific areas of high heat and heating in low-heat regions for Jeffery fluids composed of viscous and elastic materials, resulting in heterogeneous and non-uniform temperature profiles. The redistribution also depends on fluid properties such as viscosity, thermal conductivity, and elastic modulus. Optimization requires understanding the thermophoresis parameter’s effect on thermal management practices in various applications involving these fluids, such as cooling systems, industrial processes, and material processing, where accurate control of temperature is essential.

### Concentration distribution

Supplementary Figure S12 shows the variation in the concentration field of Sc. The solutal parameter varies directly with ambient mass and inversely with wall concentration. Thus, increasing the solutal parameter causes the concentration field to decrease. As a result, it can be deduced that the least value of the solutal parameter corresponds to the maximum concentration field. For instance, in chemical processing, environmental monitoring, and industrial mixing where uniform solute distribution is required, understanding phenomena like these is essential for optimizing fluid dynamics to achieve the system efficiency and product quality improvement.

Supplementary Figure S13 shows that the concentration boundary layer thins out as Sc increases. The concentration distribution in Jeffery fluid is governed by the Schmidt number, which expresses the ratio of momentum to mass diffusivity and applies to viscous–elastic fluids. A higher value of this coefficient implies that molecular diffusion is less efficient than the diffusion of momentum, which determines how a substance spreads within a liquid phase. As applied to Jeffrey liquids, changing Schmidt numbers affects the rate of diffusion and, hence, an existing concentration profile. Lower values enhance molecular diffusion for faster mixing and more uniform concentrations, while higher values impede it, causing more pronounced gradients. High accuracy becomes an essential criterion for operational output product efficiency and quality in environments such as the chemical manufacturing sector, environmental science, or biomedical technology.

An increment in the Brownian motion parameter, Nb, reduces the concentration gradient of the fluid ϕ(η) as particles move from high- to low-concentration regions, as shown in Supplementary Figure S14. The random movement of particles influenced by thermal energy makes Brownian motion influence the concentration distribution in the Jeffery fluid. Due to its randomness, this diffusion process tends to equalize concentrations over time in Jeffery fluid and has both viscous and elastic properties. Additionally, mixing is improved by Brownian motion; it also prevents particle agglutination, hence maintaining the stability of homogeneity in terms of concentration distribution. It is essential to consider the role played by Brownian motion when dealing with systems where the precise control of particle distribution, such as pharmaceuticals, chemical reactors, or materials science, is required.

The concentration field variations are treated in Supplementary Figure S15 about changes in the thermophoresis parameter (Nt). As Nt increases, the mass gradient decreases, and the thickness of the boundary layer enclosing an area of high concentration increases. A thermophoresis parameter in Jeffery fluid causes a concentration distribution that drives particles from hot to cold regions. Because this interaction is controlled by thermal gradients and viscosity and the fluid’s elasticity, it is impossible to have a uniform concentration. Through abatement or amplification, the temperature gradient reduces or increases to make a region with no equilibrium point for attaining a constant rate of heat flow; this results in non-uniformity in concentration. The thermophoretic force that arises from particle–fluid interactions and thermal conductivity makes particles gather at higher zones, thereby changing the direction of their number density contours. This behavior is essential for coatings, drug delivery systems, and pollutant dispersal applications that require accurate distribution of particles.

Supplementary Figures S16–18 present bar charts depicting the statistical analysis results. These findings support the numerical outputs derived from the variation in different parameters concerning the drag force factor, heat transference rate, and mass transference rate.

## Conclusion

This article presents a novelty analysis of the MHD Jeffrey fluid over an exponentially permeable stretching sheet, considering concentration and temperature stratification effects. Heat generation, Joule heating, viscous dissipation, and radiation are included in the study to increase the complexity of heat transfer. The problem is solved using an analytical approach, while the homotopy analysis method is applied for a sequence solution obtained. Concerning velocity, temperature, and concentration profiles, different physical parameters are analyzed.

The key findings of this analysis are as follows:

1. Increasing Deborah number values enhances velocity profiles and the thickness of the momentum boundary stratum.
2. The thermal and species fields decrease with lower thermal and concentration stratification parameters.
3. Thermophoresis and Brownian motion strictures lead to an upsurge in the temperature and width of the thermal boundary stratum.
4. Higher values of the radiative prarameter and the Eckert number result in increased temperature and thermal boundary layer thickness.
5. The results indicate an increased radial velocity distribution with higher mixed convection numbers.
6. Temperature profiles exhibit an upward trend with an increase in the Eckert number.

## Data Availability

The original contributions presented in the study are included in the article/Supplementary Material; further inquiries can be directed to the corresponding authors.

## Nomenclature

**T**: fluid temperature
**ν**: kinematic viscosity
**g**: gravitational acceleration
**V**: U, velocity components
**C**: fluid concentration
**C∞**: ambient concentration
**σ**: electrical conductivity
**ρ**: fluid density
$T_{\infty }$: ambient temperature
$T_{w}$: surface temperature
$C_{w}$: surface concentration
$C_{0}$: reference concentration
**B0**: magnetic field constant
**D**: mass diffusivity
**cp**: specific heat at constant pressure
**β**: Deborah number
**k**: thermal conductivity
**ψ**: stream function
**θ**: temperature (dimensionless)
**φ**: concentration (dimensionless).
