# Supplementary material for: Influence of dual stratification on the magnetohydrodynamic flow of Jeffrey nanofluid over an exponentially stretching permeable sheet with viscous dissipation and Joule heating
Source: Front Chem. 2025 Feb 10;12:1451053. doi: 10.3389/fchem.2024.1451053 (PMC11875100; doi:10.3389/fchem.2024.1451053)
Supplement: Supplementary file 1 [file DataSheet2.docx]

**Supplementary Figures**

**
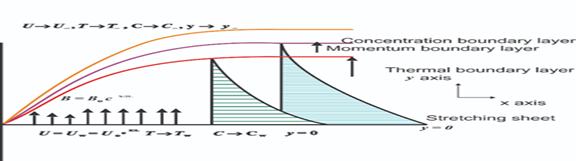
**

**Figure 1. Geometry of the problem**

**________________________________________________________________**

**
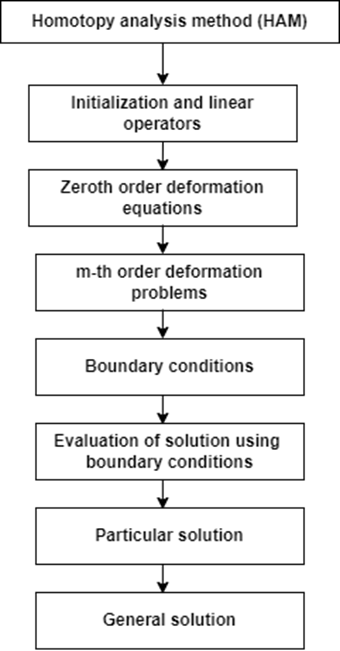
**

**Figure 2. Flow chart of HAM**

**_____________________________________________________**


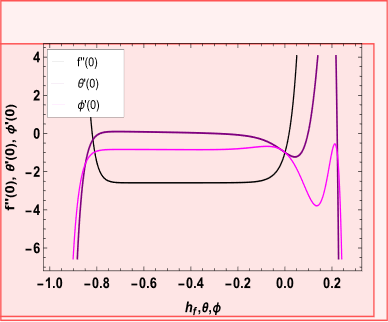


**Figure 3. h-curve for function**

**______________________________________________________________**


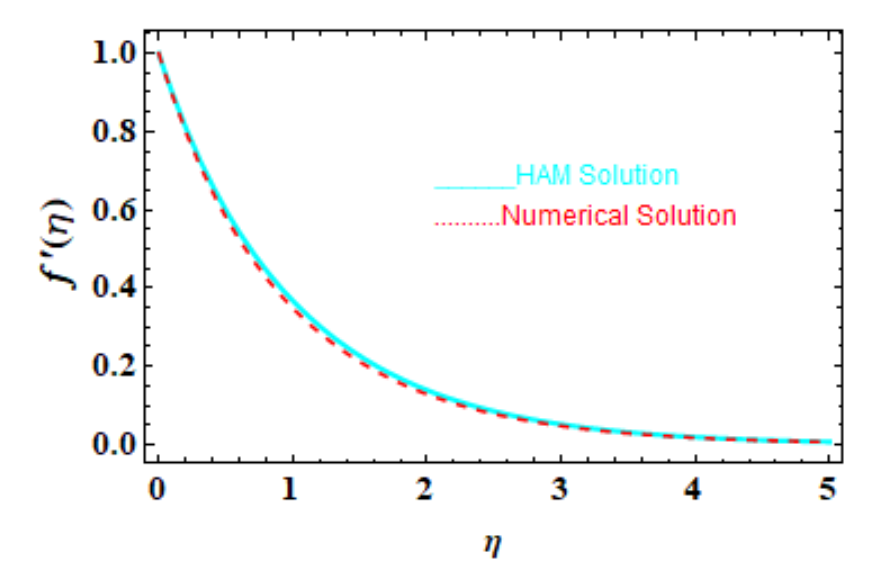


**Figure 4. HAM and numerical comparison for velocity profile** $\boldsymbol{f}^{\boldsymbol{'}}$**(η)**

**___________________________________________________________**

**
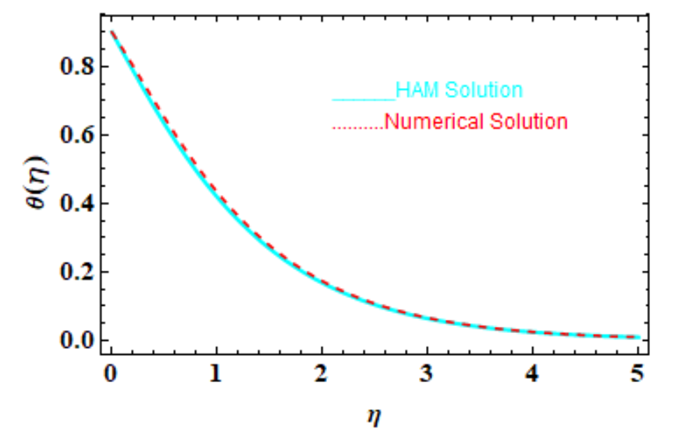
**

**Figure5. HAM and numerical comparison for temperature profile θ(η)**

**
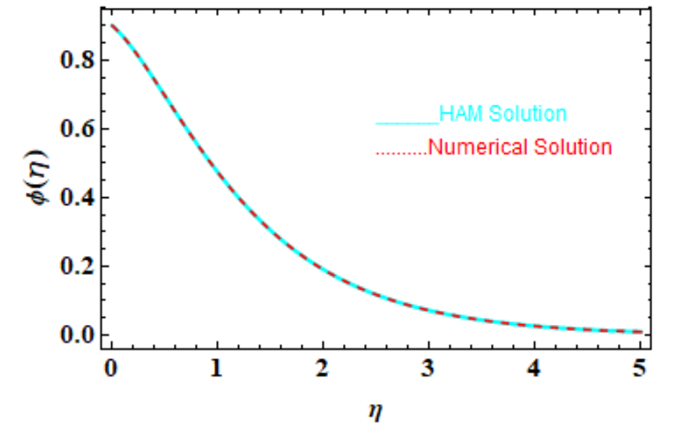
**

**Figure 6. HAM and numerical comparison for concentration profile** $\boldsymbol{ɸ}$**(η)**


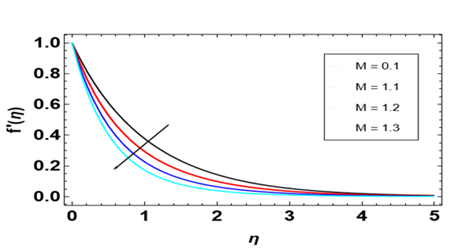


**Figure 7. The influence of M versus** $\boldsymbol{f}^{\boldsymbol{'}}$**(η)**

**_______________________________________________________________**


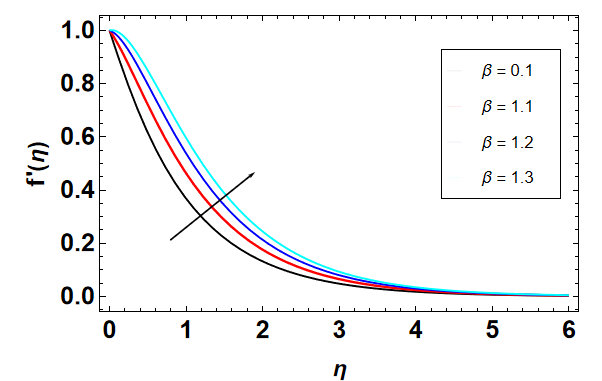


**Figure 8. The influence of β versus** $\boldsymbol{f}^{\boldsymbol{'}}$**(η)**

**________________________________________________________________**

**
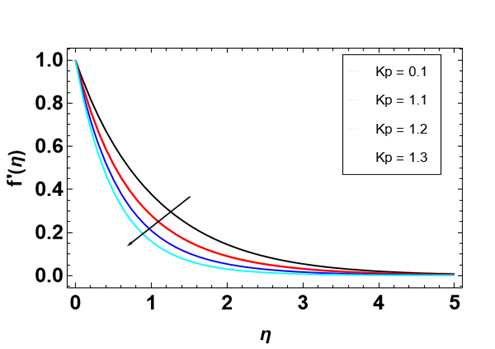
**

**Figure 9. The influence of Kp versus**$\boldsymbol{f}^{\boldsymbol{'}}$**(η)**

**_________________________________________________________**


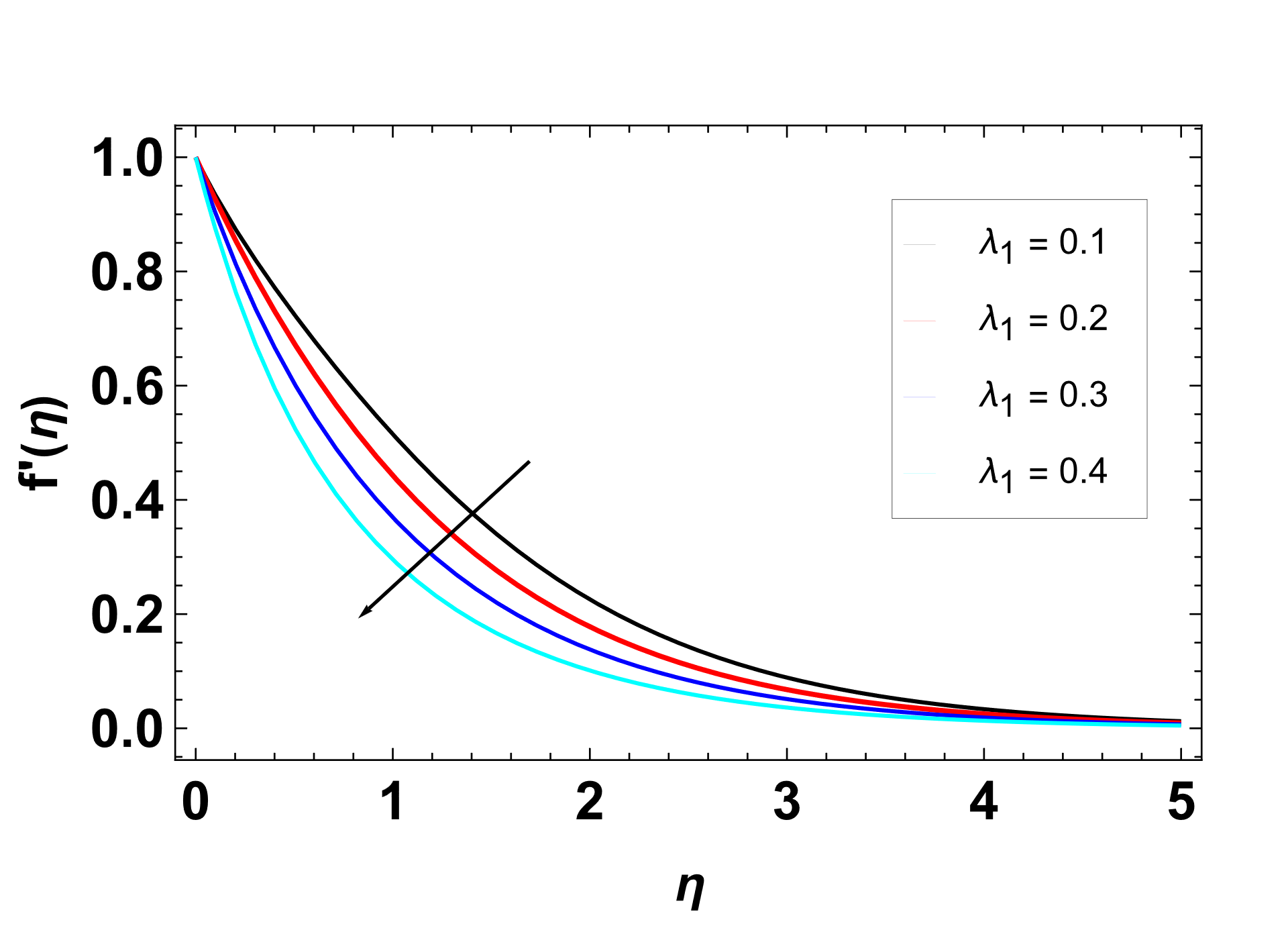


**Figure 10. The influence of** $\boldsymbol{\lambda}_{\boldsymbol{1}}$ **versus** $\boldsymbol{f}^{\boldsymbol{'}}$**(η)**

**________________________________________________________________**

**
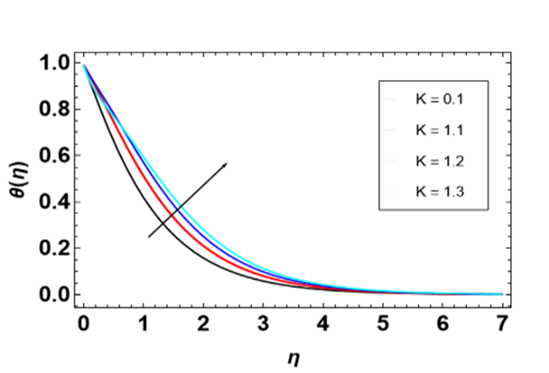
**

**Figure 11. The influence of K versus θ(η)**

**_________________________________________________________**

**
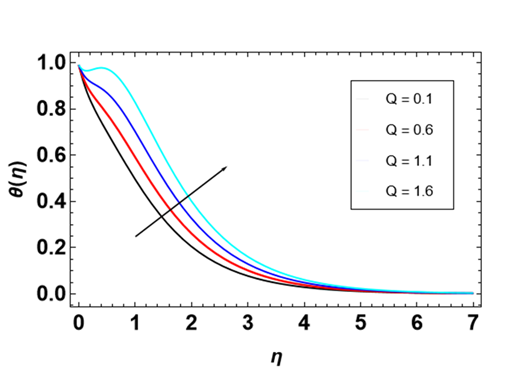
**

**Figure 12. The influence of Q versus θ(η)**

**________________________________________________________________**

**
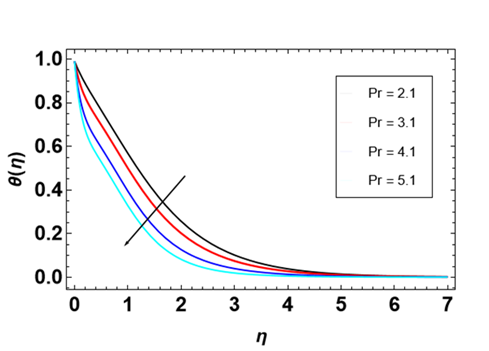
**

**Figure 13. The influence of Pr versus θ(η)**

**
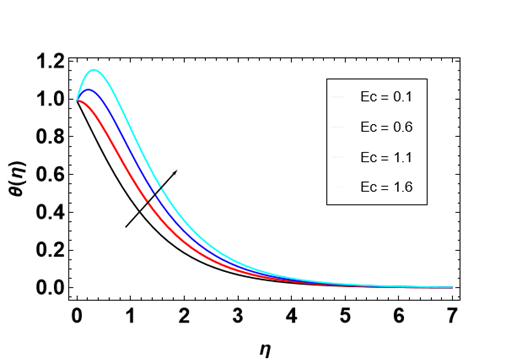
**

**Figure 14. The influence of Ec versus θ(η)**

**____________________________________________________________**

**
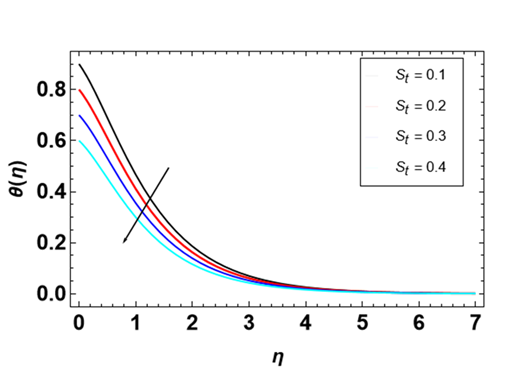
**

**Figure15.The influence of** $\boldsymbol{S}_{\boldsymbol{t}}$ **versus θ(η)**

**_________________________________________________________**

**
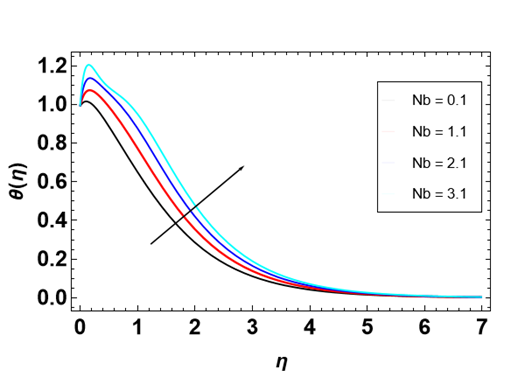
**

**Figure16.The influence of Nb versus θ(η)**

**__________________________________________________________**

**
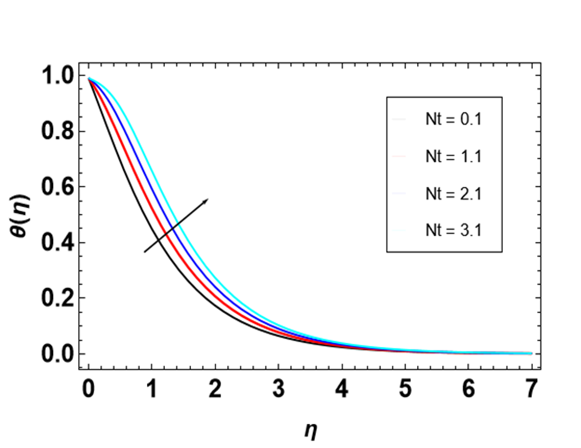
**

**Figure 17. The influence of Nt versus θ(η)**

**___________________________________________________________________**

**
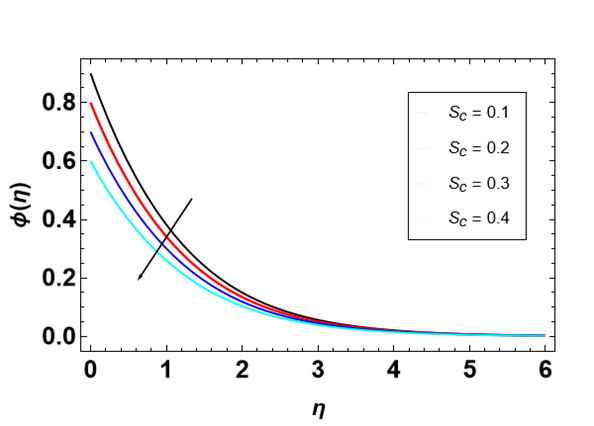
**

**Figure 18. The influence of** $\boldsymbol{S}_{\boldsymbol{c}}$ **versus**$\boldsymbol{ɸ}$**(η)**

**________________________________________________________________**

**
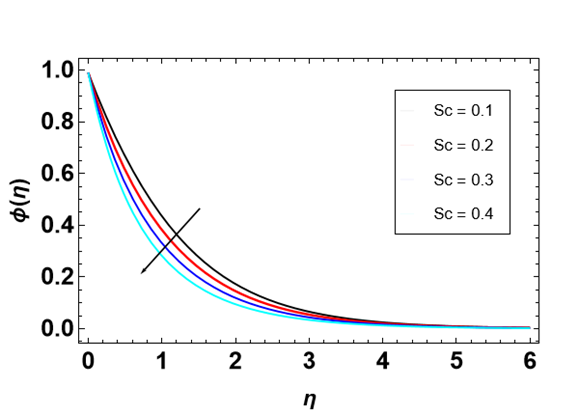
**

**Figure 19. The influence of Sc versus** $\boldsymbol{ɸ}$**(η)**

**_______________________________________________________**

**
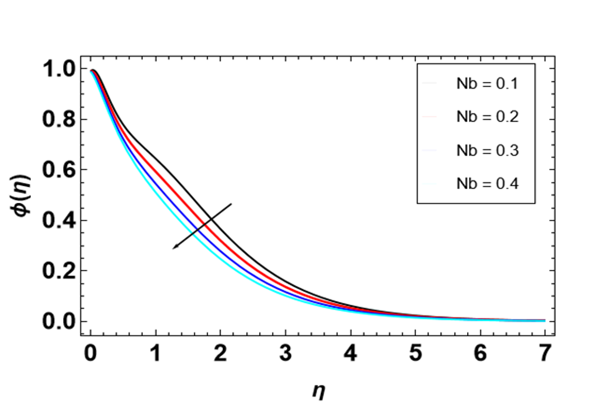
**

**Figure 20. The influence of Nb versus** $\boldsymbol{ɸ}$**(η)**

**__________________________________________________________**

**
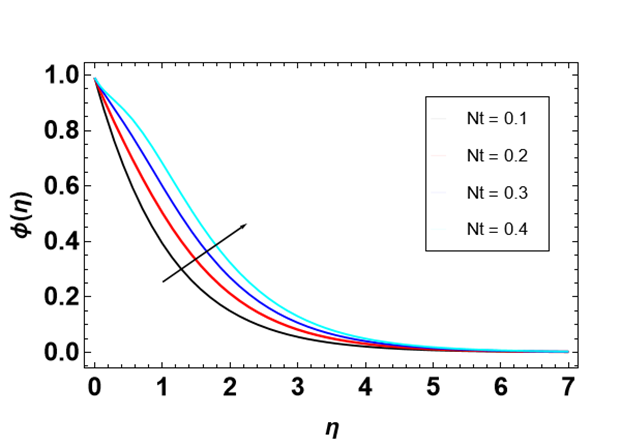
**

**Figure 21. The influence of Nt versus** $\boldsymbol{ɸ}$**(η)**

**_________________________________________________________**

**
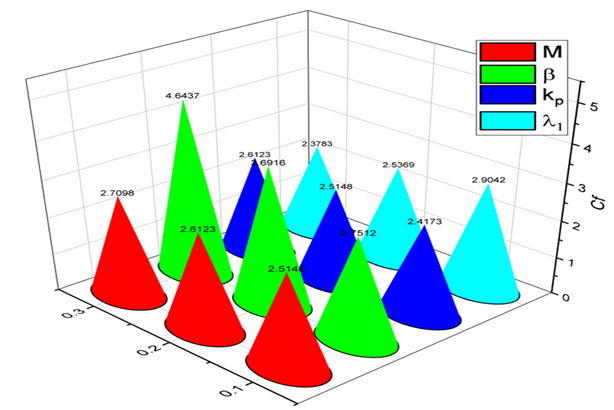
**

**Figure 22. Skin friction versus M, β,**$\boldsymbol{\lambda}_{\boldsymbol{1}}$ **and Kp**

**_________________________________________________________**

**
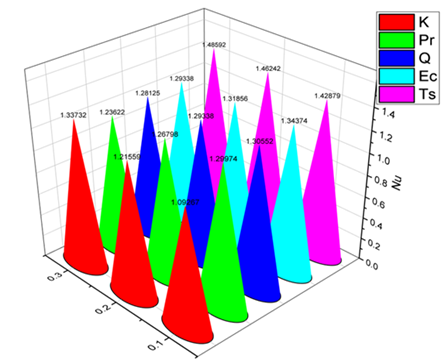
**

**Figure 23. The heat transfer rate versus K, Pr, Q, Ec, Ts**.

___________________________________________________________________

**
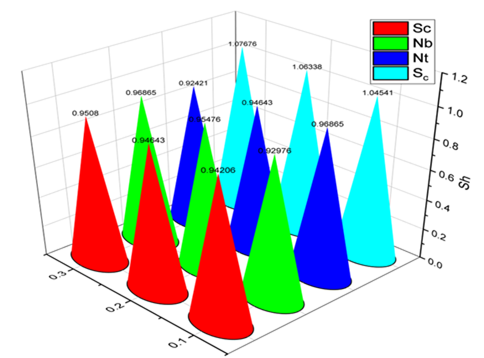
**

**Figure 24. The mass transfer rate versus Sc, Nb, Nt,** $\boldsymbol{S}_{\boldsymbol{c}}$ **.**

**________________________________________________________________**
