# Supplementary material for: Influence of dual stratification on the magnetohydrodynamic flow of Jeffrey nanofluid over an exponentially stretching permeable sheet with viscous dissipation and Joule heating
Source: Front Chem. 2025 Feb 10;12:1451053. doi: 10.3389/fchem.2024.1451053 (PMC11875100; doi:10.3389/fchem.2024.1451053)
Supplement: Supplementary file 2 [file DataSheet1.docx]

**Supplementary Tables**

_____________________________________________________________________

**Table 1. HAM solutions convergence at various approximation order**

| M | $f''(0)$ | $\theta'(0)$ |  |
| --- | --- | --- | --- |
| 4 | $-1.22139$ | $-0.78096$ | $-0.707819$ |
| 8 | $-1.3250$ | $-0.693755$ | $-0.584240$ |
| 12 | $-1.37449$ | $-0.628956$ | $-0.50381$2 |
| 16 | $-1.39876$ | $-0.579765$ | $-0.450614$ |
| 20 | $-1.4113$ | $-0.549421$ | $-0.419513$ |
| 24 | $-1.42952$ | $-0.526218$ | $-0.396782$ |
| 28 | $-1.43835$ | $-0.507231$ | $-0.370321$ |
| 32 | $-1.44952$ | $-0.496429$ | $-0.358951$ |
| 36 | $-1.45126$ | $-0.489532$ | $-0.349825$ |
| 40 | $-1.45334$ | $-0.487539$ | $-0.337423$ |
| 44 | $-1.45426$ | $-0.486943$ | $-0.336732$ |
| 48 | $-1.45432$ | $-0.486694$ | $-0.336087$ |
| 50 | $-1.45432$ | $-0.486694$ | $-0.336087$ |

______________________________________________________________________

**Table 2. HAM and numerical comparison for velocity profile $f'(\eta)$**

| η | HAM solution | Numerical solution | Absolute Error |
| --- | --- | --- | --- |
| 0.0 | 1.000000 | 1.000000 | 0.000000 |
| 0.5 | 0.599264 | 0.598658 | 0.001213 |
| 1.0 | 0.367317 | 0.366674 | 0.001286 |
| 1.5 | 0.226250 | 0.225743 | 0.001013 |
| 2.0 | 0.139053 | 0.138699 | 0.000709 |
| 2.5 | 0.085149 | 0.084916 | 0.000466 |
| 3.0 | 0.051976 | 0.051827 | 0.000297 |
| 3.5 | 0.031654 | 0.031561 | 0.000185 |
| 4.0 | 0.019248 | 0.019191 | 0.000114 |
| 4.5 | 0.011693 | 0.011658 | 0.000070 |
| 5.0 | 0.007099 | 0.007078 | 0.000043 |

___________________________________________________________________________

**Table 3. HAM and numerical comparison for temperature profile $\theta(\eta)$**

| η | HAM solution | Numerical solution | Absolute Error |
| --- | --- | --- | --- |
| 0.0 | 0.900000 | 0.900000 | 0.000000 |
| 0.5 | $0.631487$ | $0.632116$ | $0.001257$ |
| 1.0 | $0.417822$ | $0.418306$ | $0.000968$ |
| 1.5 | $0.267020$ | $0.267320$ | $0.000600$ |
| 2.0 | $0.167141$ | $0.167318$ | $0.000353$ |
| 2.5 | $0.103325$ | $0.103428$ | $0.000206$ |
| 3.0 | $0.063396$ | $0.063456$ | $0.000122$ |
| 3.5 | $0.038720$ | $0.038757$ | $0.000072$ |
| 4.0 | $0.023585$ | $0.023606$ | $0.000043$ |
| 4.5 | $0.014342$ | $0.014354$ | $0.000026$ |
| 5.0 | $0.008712$ | $0.008720$ | $0.000016$ |

_____________________________________________________________________

`**Table 4. HAM and numerical comparison for concentration profile**

| $\eta$ | HAM solution | Numerical solution | Absolute Error |
| --- | --- | --- | --- |
| $0.0$ | $0.900000$ | $0.900000$ | $0.000000"$ |
| $0.5$ | $0.695182$ | $0.900000$ | $0.000010"$ |
| $1.0$ | $0.472915$ | $0.472917$ | $.47\times{10}^{-6}$ |
| $1.5$ | $0.304204$ | $0.472917$ | $1.59\times{10}^{-6}$ |
| $2.0$ | $0.190448$ | $0.190448$ | $6.11\times{10}^{-7}$ |
| $2.5$ | $0.117579$ | $0.117579$ | $2.76\times{10}^{-7}$ |
| $3.0$ | $0.072047$ | $0.072047$ | $1.44\times{10}^{-7}$ |
| $3.5$ | $0.043961$ | $0.043961$ | $8.23\times{10}^{-8}$ |
| $4.0$ | $0.026759$ | $0.026759$ | $4.87\times{10}^{-8}$ |
| $4.5$ | $0.016264$ | $0.016264$ | $2.93\times{10}^{-8}$ |
| $5.0$ | $0.009878$ | $0.009878$ | $1.77\times{10}^{-8}$ |

__________________________________________________________________________

**Table 5: Numerical outcomes of** $\boldsymbol{Cf}_{\boldsymbol{x}}$ **for parameters M, β,**$\boldsymbol{\lambda}_{\boldsymbol{1}}$ **and Kp**

| M | β | Kp | $\lambda_{1}$ | $f''(0)$ |
| --- | --- | --- | --- | --- |
| 1 |  |  |  | 2.5148 |
| 1.5 |  |  |  | 2.6123 |
| 2 |  |  |  | 2.7098 |
|  | 0.5 |  |  | 2.7512 |
|  | 1 |  |  | 3.6916 |
|  | 1.5 |  |  | 4.6437 |
|  |  | 0.5 |  | 2.4173 |
|  |  | 1 |  | 2.5148 |
|  |  | 1.5 |  | 2.6123 |
|  |  |  | 1 | 2.9042 |
|  |  |  | 1.5 | 2.5369 |
|  |  |  | 2 | 2.3783 |

**Table 6: Numerical outcomes of** $\boldsymbol{Nu}_{\boldsymbol{x}}$ **for parameters K, Pr, Q, Ec and Ts**

| K | Pr | Q | Ec | Ts | $\theta'(0)$ |
| --- | --- | --- | --- | --- | --- |
| 0.1 |  |  |  |  | 1.09267 |
| 0.2 |  |  |  |  | 1.21559 |
| 0.3 |  |  |  |  | 1.33732 |
|  | 1.1 |  |  |  | 1.29974 |
|  | 2.1 |  |  |  | 1.26798 |
|  | 3.1 |  |  |  | 1.23622 |
|  |  | 0.1 |  |  | 1.30552 |
|  |  | 0.3 |  |  | 1.29338 |
|  |  | 0.5 |  |  | 1.28125 |
|  |  |  | 0.1 |  | 1.34374 |
|  |  |  | 0.2 |  | 1.31856 |
|  |  |  | 0.3 |  | 1.29338 |
|  |  |  |  | 0.1 | 1.42879 |
|  |  |  |  | 0.2 | 1.46242 |
|  |  |  |  | 0.3 | 1.48592 |

__________________________________________________________________

**Table 7: Numerical outcomes of** $\boldsymbol{Sh}_{\boldsymbol{x}}$ **for parameters SC, Nb, Nt and Sc.**

| SC | Nb | Nt | Sc | $ɸ'(0)$ |
| --- | --- | --- | --- | --- |
| 0.2 |  |  |  | 0.94206 |
| 0.3 |  |  |  | 0.94643 |
| 0.4 |  |  |  | 0.95080 |
|  | 0.2 |  |  | 0.92976 |
|  | 0.4 |  |  | 0.95476 |
|  | 0.6 |  |  | 0.96865 |
|  |  | 0.1 |  | 0.96865 |
|  |  | 0.3 |  | 0.94643 |
|  |  | 0.5 |  | 0.92421 |
|  |  |  | 0.1 | 1.04541 |
|  |  |  | 0.2 | 1.06338 |
|  |  |  | 0.3 | 1.07676 |

_________________________________________________________________________
